# Microbial translocation is correlated with HIV evolution in HIV-HCV co-infected patients

**DOI:** 10.1371/journal.pone.0183372

**Published:** 2017-09-21

**Authors:** Jean-Jacques Tudesq, Catherine Dunyach-Remy, Christophe Combescure, Régine Doncesco, Didier Laureillard, Jean-Philippe Lavigne, Albert Sotto

**Affiliations:** 1 Institut National de la Santé et de la Recherche Médicale, Montpellier University, Nîmes, France; 2 Department of Microbiology, Nîmes University Hospital, Nîmes, France; 3 Division of Clinical epidemiology, Department of Health and Community Medicine, University of Geneva and University Hospitals of Geneva, Geneva, Switzerland; 4 Department of Infectious diseases, Nîmes University Hospital, Nîmes, France; University of Pittsburgh Centre for Vaccine Research, UNITED STATES

## Abstract

Microbial translocation (MT) is characterized by bacterial products passing into the blood through the gut barrier and is a key phenomenon in the pathophysiology of Human Immunodeficiency Virus (HIV) infection. MT is also associated with liver damage in Hepatitis C Virus (HCV) patients. The aim of the study was to assess MT in plasma of HIV-HCV co-infected patients. 16S rDNA (16 S Ribosomal DNA subunit) marker and other markers of MT such as Lipopolysaccharide (LPS)-binding protein (LBP), soluble CD14 (sCD14), intestinal fatty acid binding protein (I-FABP) were used. Clinical, biological and immunological characteristics of the population were studied in order to correlate them with the intensity of the MT. We demonstrate that indirect markers of MT, LBP and CD14s, and a marker of intestinal permeability (I-FABP) are significantly higher in HIV-HCV co-infected patients than in healthy controls (17.0 vs 2.6 μg/mL, p < 0.001; 1901.7 vs 1255.0 ng/mL, p = 0.018); 478.3 vs 248.1 pg/mL, p < 0.001, respectively), while a direct marker of MT (16S rDNA copies) is not different between these two populations. However, plasma 16S rDNA was significantly higher in co-infected patients with long-standing HIV infections (RGM = 1.47 per 10 years, CI95% = [1.04:2.06], p = 0.03). Our findings show that in HIV-HCV co-infected patients, plasma 16S rDNA levels, directly reflecting MT, seem to be linked to the duration of HIV infection, while elevated levels of LBP and sCD14 reflect only a persistence of immune activation. The levels of these markers were not correlated with HCV evolution.

## Introduction

Microbial translocation (MT) is a feature in people living with HIV, and is associated with immune activation, a negative prognosis factor in HIV infection [1–8]. MT is thought to be associated with loss of mucosal barrier function, persistent viral replication in the gut and increased intestinal permeability resulting from immune deregulation and alterations in the intestinal microbiome [9]. In contrast, efficient anti-retroviral therapy (ART) in HIV mono-infected efficient anti-retroviral therapy (ART) in HIV mono-infected patients leads to a reduction of MT, although immune activation persists [5,7]. This persistent innate and acquired immune activation contributes to the pathogenesis of non-AIDS-related diseases [2]. Immune activation has also been described in hepatitis C virus (HCV) co-infections. Indeed, in HIV-HCV co-infected patients, this immune activation promotes HCV hepatic fibrosis progression, resulting in poorer prognosis compared to HIV mono-infected patients, even in ART-controlled HIV [10–12].

Previous studies have shown that MT is associated with uncontrolled HIV infection as well as liver damage in HCV patients, but few studies have analyzed MT in HIV-HCV co-infected patients [13, 14], particularly taking into account the clinical and biological parameters of these patients. The aim of this study was to assess MT in HIV-HCV co-infected patients using a direct marker (plasmatic ribosomal DNA subunit 16 (16s rDNA)) and indirect markers of MT, reflecting immune host response (soluble cluster of differentiation 14 (sCD14), LPS-binding protein (LBP)) and a marker of intestinal permeability (I-FABP) to correlate these markers with clinical, biological and immunological characteristics of co-infected patients.

## Materials and methods

### Patients and controls

Plasma samples from 145 HIV-HCV co-infected patients undergoing regular follow-up at Nîmes University Hospital between September 2012 and September 2014 were analyzed. Additionally, plasma from 100 healthy blood donors from Etablissement Français du Sang were analyzed as a control group. Included patients had no infectious disease within the 15 days prior to and following the blood sample. Routine blood parameters were performed on these samples including liver function tests, CD4+T-cell count and HIV RNA viral loads (VL). Demographic, clinical and immunological data concerning HCV genotype and fibrosis assessment were collected from medical records.

### Bacterial translocation markers

#### DNA extraction

Plasma samples were collected in EDTA-anticoagulated tubes and stored at -80°C. DNA was extracted from 200 μL of plasma using the EZ1 DNA Tissue kit (Qiagen, Courtaboeuf, France) according to the manufacturer’s instructions. DNA was eluted in a 100 μL final volume.

#### 16S rDNA real-time PCR

Bacterial 16S rDNA levels were measured by qPCR. A 20 μL amplification reaction consisted of 4 μL of *LightCycler FastStart DNA Master*^*PLUS*^ *HybProbe* (Roche, Meylan, France), 0.5 μmol/L forward and reverse primers, 1 μmol/L TaqMan probe and 10 μL of the template plasma DNA. Degenerate forward (16S-F: 5'-AACAGGATTAGATACCCTGGTAG-3' nucleotide 780 to 802) and reverse (16S-R: 5'-GGTTCTKCGCGTTGCWTC-3' nucleotide 962 to 979) primers were used to amplify the hypervariable region V5 of the 16S gene. Carboxyfluorescein stained probe (16S-probe: 5’-FAM-AACAC5TGCTCCACCGCT-BHQ1-3’ nucleotide 937 to 948, where FAM means 6-carboxyfluorescein and BHQ1 means Black Hole Quencher I) was used as described by Kramski *et al*. [15]. The amplified region was 199bp. The DNA was independently amplified twice in duplicate, and mean values were calculated. A negative control (molecular biology grade water) was systematically used. A standard curve was created from serial dilutions of plasmid DNA containing known copy numbers of the template. The reaction conditions for amplification of DNA were 95°C for 10 min, followed by 45 cycles at 95°C for 15 s, 60°C for 60 s. The assays were performed using a LightCycler 480 II (Roche), with absolute quantification analysis performed with the Lightcycler 480 software (Roche), version 1.5, according to manufacturer’s recommendations.

#### sCD14, LBP and I-FABP

LBP, sCD14 and I-FABP levels were measured by ELISA test. LBP plasma level was measured using Enzyme Immunoassay for Quantification of free human LBP ELISA kit (Enzo Life Sciences, Villeurbanne, France), with a dilution factor of 1:800, according to manufacturer’s recommendations, and sCD14 plasma level was measured using Quantikine ELISA Human sCD14 kit (R&D Systems, Lille, France), according to manufacturer’s specifications. Samples were diluted 1:200 except for five, which were diluted 1:500 because of their higher concentration. I-FABP plasma level was measured using Human I-FABP ELISA kit (Hycult Biotech, Uden, the Netherlands) according to manufacturer’s recommendations and a dilution factor of 1:2.

### Statistical analysis

Patients’ characteristics and plasma levels are presented as median ± interquartile range (IQR), geometric mean (95% IC) or number and percentage. Correlations between 16S rDNA and LPS, sCD14, or LBP levels were assessed with Spearman’s correlation coefficients. Unadjusted associations between plasma levels and patients’ characteristics were tested using t tests. Adjusted associations were assessed using linear regression models with CDC stage, detectable viral load, CD4<350 cells/μL (immunosuppression) and duration of the HIV infection as independent variables. Age and gender were introduced into the model for adjustment. Since the plasma 16srDNA, sCD14 and I-FABP levels were log-normally distributed, they were log-transformed for statistical analyses. Regression coefficients were expressed as ratio of geometric mean (RGM). All statistical tests were two-sided with a significance level of 0.05. Statistical analyses were performed with S-plus 8.0 for Windows (Insightful Corp., Seattle, WA, USA).

### Ethical aspects

The study protocol was approved by the Institutional Review Board of University Hospital, Nîmes, France (N°14/12.0) and conformed to the ethical guidelines of the Declaration of Helsinki. Written informed consent was obtained from each participant.

## Results and discussion

### Study population

Baseline characteristics of the 145 patients (43 women) included in the study are summarized in Table 1. The median age of those enrolled was 52 years [48:55]. Most patients (91.7%) were receiving ART. The median CD4+T-cell count was 468/μL and 74.5% had an undetectable HIV viral load. Among the 145 patients, 120 had a chronic hepatitis C, 22 had a spontaneously resolved infection and 3 had an acute infection. Half of the patients (56.3%) were infected with HCV-1 genotype and a quarter (25.0%) with HCV-3, which is associated with a better prognosis [16]. Of the 112 patients with a fibrosis (METAVIR) score, nearly 30% were classified as F3-F4. A total of 65 patients had received anti-HCV therapy (PEG-interferon and ribavirine): 47.7% were responders, 20.0% non-responders, 6.2% partial responders and 13.8% relapsers.

**Table 1:** Baseline characteristics of study population.

Variable		N (%) or median [interquartile range]
**Sex-ratio (n = 145)**	Women	29.7%
**Age**	Years	52 [48:55]
**CDC**^**a**^ **clinical stage (n = 144)**	A	52.1%
	B	25.0%
** **	C	22.9%
**Duration of HIV infection (n = 144)**	Years	21.9 [17.5:26.6]
**ART (n = 145)**	Yes	91.7%
**HIV viral load (n = 145)**	<20 copies/mL	74.5%
**if detectable**	copies/mL	306 [54:12100]
**CD4 T cells count (n = 144)**	cells/μL	468 [317:634]
**<350 cells/**μ**L**	Yes	29.2%
**HCV genotype (n = 112)**	1	56.3%
	2	2.7%
	3	25.0%
	4	16.1%
**HCV viral load (n = 138)**	copies/mL	188721 [0:1885000]
**Type of HCV infection (n = 145)**	Chronic infection	82.8%
	Spontaneously resolved	15.2%
** **	Acute	2.1%
**Outcome after anti-HCV therapy (n = 145)**	Sustained VR ^b^	47.7%
	Partial VR	6.2%
	Relapse	13.8%
	No response	20.0%
	Discontinued treatment	7.7%
	In treatment	4.6%
** **	Not applicable (not treated)	55%
**HBV co-infection (n = 145)**	Yes	12.4%
**METAVIR score**	(Only if chronic: n = 120)	
**Assessment method (n = 113)**	Liver biopsy	20.4%
	Fibroscan®	77.9%
	Actitest-Fibrotest®	1.8%
**Score (n = 137)**	F0 or F1	54.5%
	F2	16.1%
	F3	11.6%
	F4	17.9%
**Plasma 16S rDNA (n = 145)**	copies/μL	9.17^f^ (7.79:10.8)
**Plasma LBP**^c^ **(n = 132)**	μg/mL	17.0^f^ (15.5:18.6)
**Plasma sCD14**^d^**(n = 132)**	ng/mL	1901.7 [1573.2:2232.8]
**Plasma I-FABP**^e^ **(n = 132)**	pg/mL	478.3^f^ (412.5:554.5)

^a^Centers for Disease Control and Prevention.

^b^Virologic response

^c^Lipopolysaccharide (LPS)-binding protein

^d^soluble CD14

^e^ intestinal fatty acid binding protein

^f^Geometric mean (95% IC)

### LPB, sCD14 and I-FABP markers but not 16S rDNA were significantly higher in HIV-HCV co-infected patients than in healthy controls

Geometric mean (95%IC) or median (IQR) plasma levels of 16S rDNA, LBP, sCD14 and I-FABP in co-infected patients were 9.17 copies/μL (95%IC (7.79:10.8)), 17.0 μg/mL (95%IC (15.5:18.6)), 1901.7ng/mL (IQR [1573.2:2232.8]) and 478.3 pg/mL (95%IC (412.5:554.5)) respectively.

**Fig 1:**
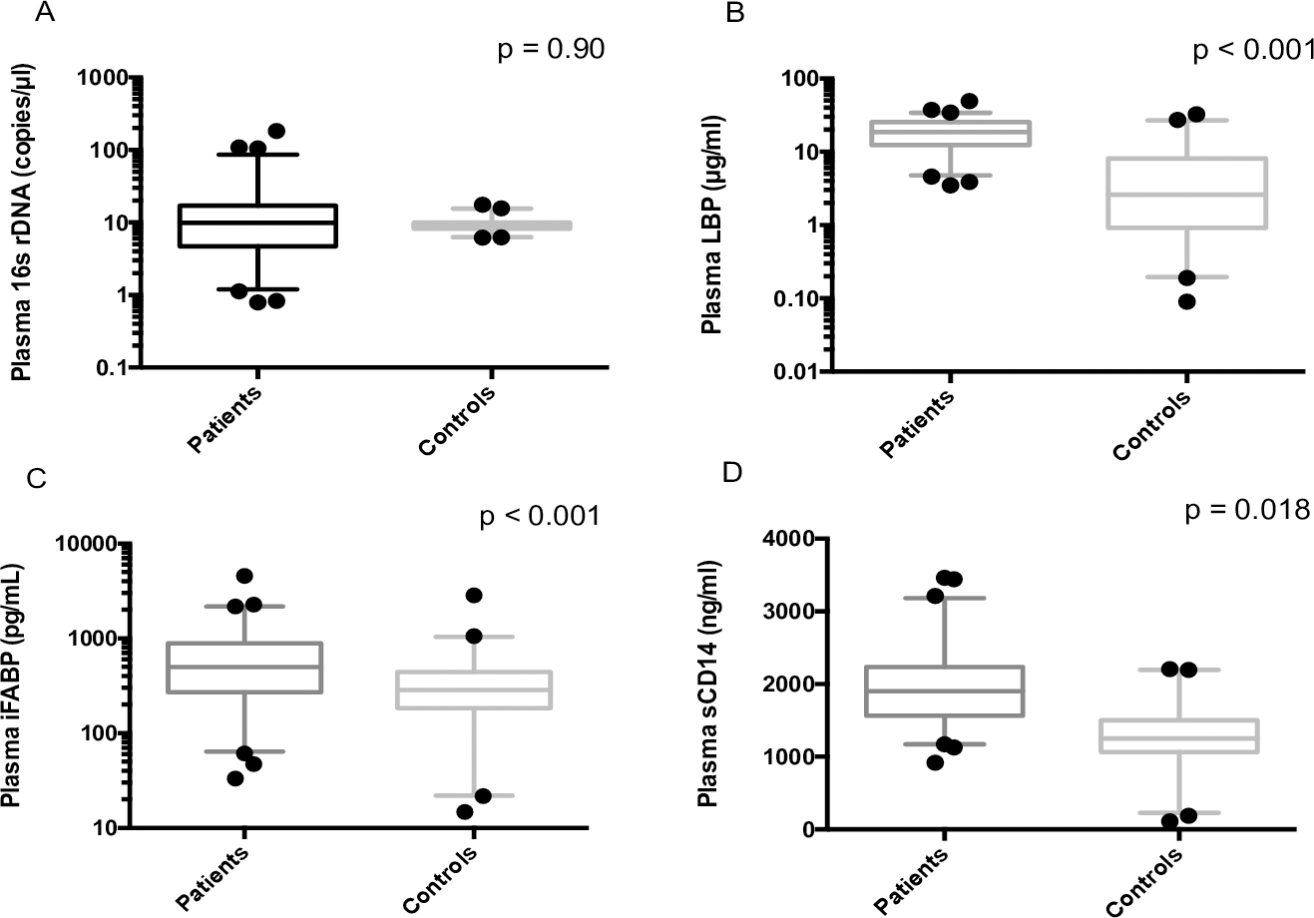
MT markers comparison between co-infected HIV-HCV patients and healthy controls. Markers of the immune response such as LBP or sCD14 and intestinal permeability marker I-FABP were significantly higher in HIV-HCV co-infected patients than in healthy controls. The number of 16s rDNA copies is comparable in the two populations (p = 0.90).

The number of 16S rDNA copies was not significantly different between co-infected patients and healthy controls (9.173 vs 9.074 copies/μL, p = 0.90) (Fig 1). These rates are similar to those published in ART-controlled HIV mono-infected patients [3, 4, 5, 15]. Moreover, this result is in accordance with several previously published works, where MT is only associated with uncontrolled HIV infection or with a severe hepatic burden (cirrhosis) [5, 15, 17]. However, Sacchi *et al*. have shown that levels of bacterial DNA were significantly higher in HIV-HCV co-infected group than in an ART-controlled HIV-positive group [13].

LBP, sCD14 and I-FABP markers were significantly higher in HIV-HCV co-infected patients than in healthy controls (17.0 vs 2.6 μg/mL, p < 0.001; 1901.7 vs 1255.0 ng/mL, p = 0.018; 478.3 vs 248.1 pg/mL, p < 0.001, respectively) **(Fig 1).** Results for LBP and sCD14 were comparable to those obtained by Nystrom *et al*., reflecting persistence of immune activation in these patients rather than translocation of bacterial products [14]. Moreover, other studies have demonstrated that the rates of I-FABP were higher in HIV patients than in HIV-uninfected patients [18] and that levels of I-FABP were comparably increased in HIV mono-infection and HIV-HCV co-infection [19].

The associations between MT markers were analyzed for all 145 plasma samples. No correlation was found between 16S rDNA and LPS, sCD14, or LBP levels with Spearman’s correlation coefficients (ρ = -0.25 p = 0.004; ρ = 0.16 p = 0.07; and ρ = 0.00 p = 1, respectively). This result was in accordance with the work published by Abad-Fernandez *et al*., who compared several MT markers in HIV mono-infected patients [3]. However, other studies contradict these findings, instead describing correlation between LPS and 16S rDNA [5]. It is important to note that bacterial 16S rDNA was directly measured by qPCR, whilst LBP, sCD14 and I-FABP levels only indirectly indicate presence of MT.

### In HIV-HCV co-infected patients, plasma 16S rDNA increased with the duration of the HIV infection

**Fig 2:**
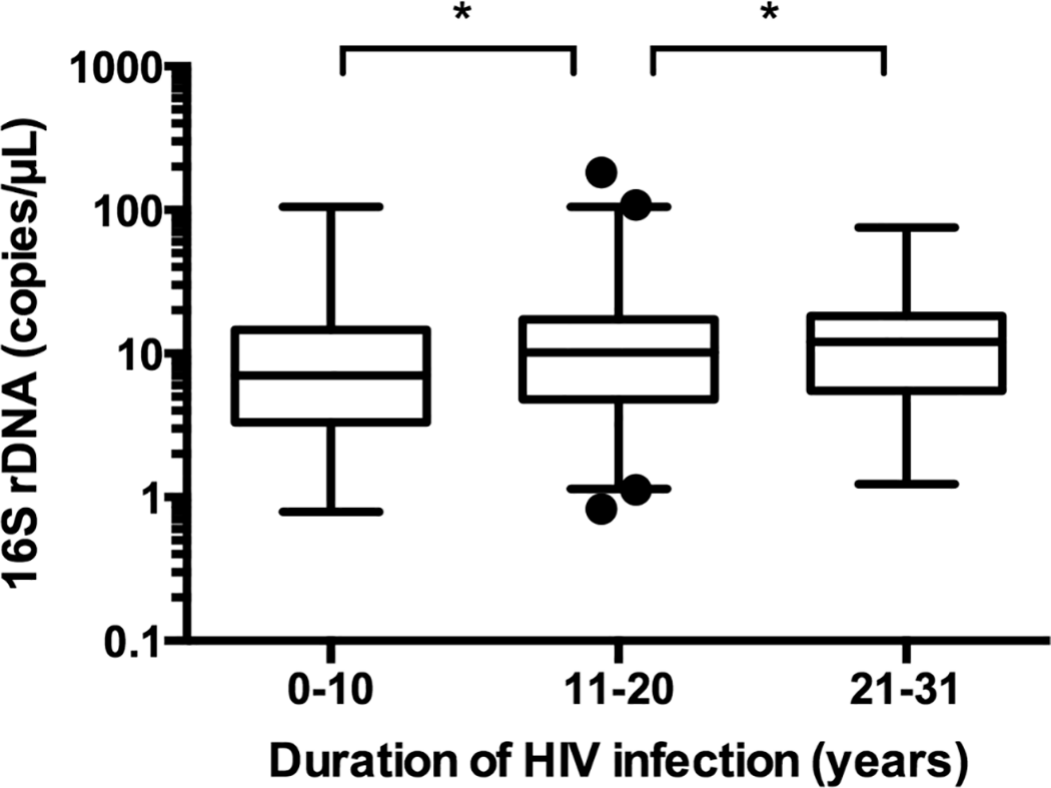
Microbial translocation assessed by plasma 16S rDNA in HIV-HCV co-infected patients. Microbial translocation assessed by plasma 16S rDNA increases with the duration of HIV infection. *: p < 0.05 (Student t test).

**Fig 3:**
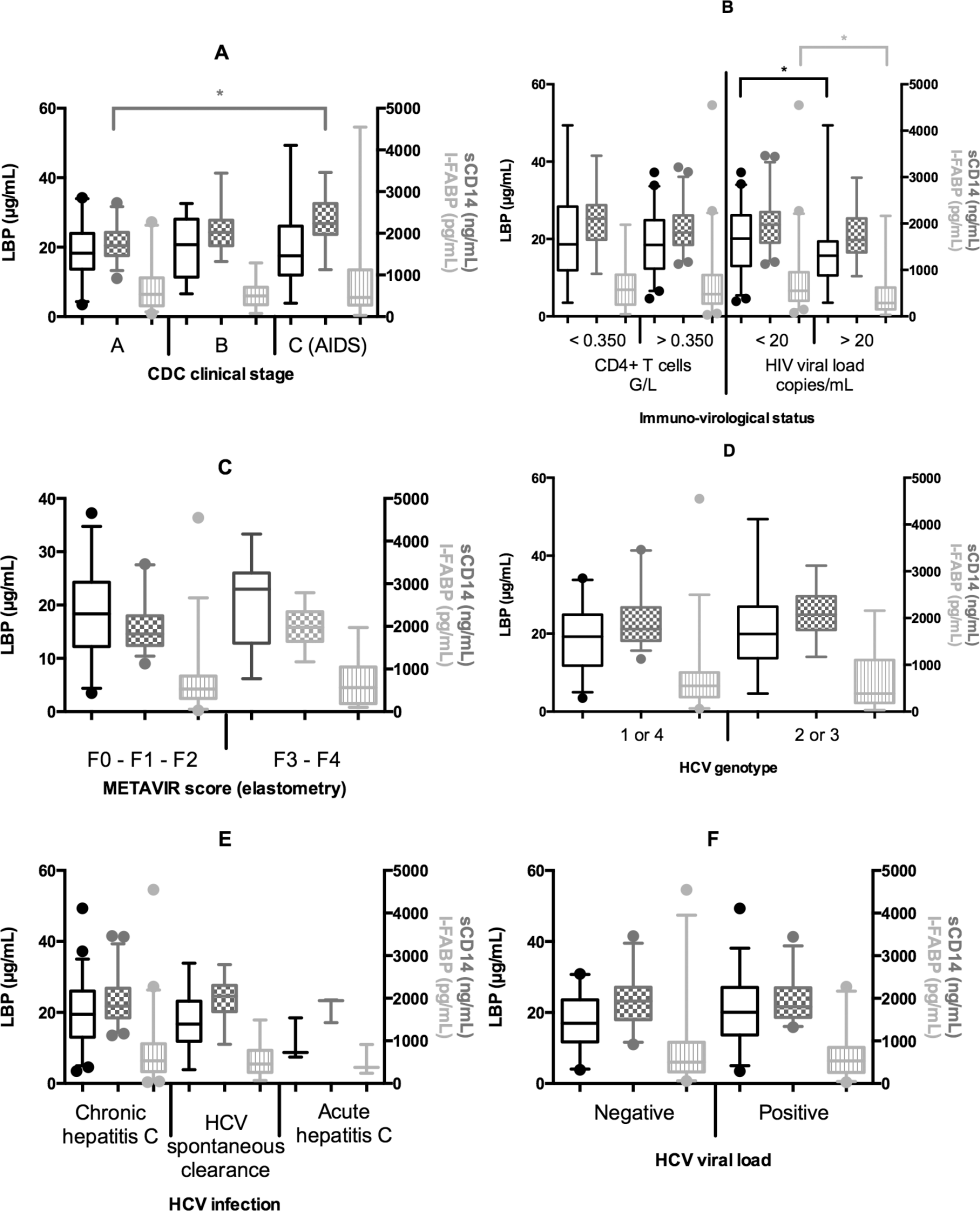
**Plasma LBP (Lipopolysaccharide-binding protein) (black), sCD14 (soluble Cluster Differentiation 14) (dark gray) and I-FABP (intestinal fatty acid binding protein) (light gray) according to:** (A) CDC clinical stage of the HIV infection; (B) immuno-virological status of the HIV infection; (C) METAVIR fibrosis score; (D) HCV genotype; (E) HCV infection type and (F) HCV viral load. *: p < 0.05 (Student t test).

Univariate analysis showed that plasma 16S rDNA increased with the duration of the HIV infection (p = 0.047) (Fig 2). However, plasma 16S rDNA was not associated with the CDC stage, the HIV viral load, the CD4+ cells count, the detectable HIV viral load, the HCV genotype, HCV viral load, or the METAVIR score. The multivariate linear regression analyses confirmed the findings of the univariate comparisons. Plasma 16S rDNA was significantly higher in co-infected patients with long-standing HIV infections (RGM = 1.47 per 10 years, CI95% = [1.04:2.06], p = 0.03). This study showed, for the first time, a correlation between the duration of HIV infection and the level of bacterial translocation in HIV-HCV co-infected patients. This could be explained by the fluctuating viral loads in long-term infected patients, with periods of suboptimal control leading to MT resurgence. However, Baroncelli *et al*. described that repeated ART interruptions were not associated with plasma LPS elevation [20]. Another explanation was that MT was linked to the HIV reservoir and the residual viremia (viral replication under 50 copies/mL) in HIV controlled patients. This hypothesis would explain the persistent immune activation in ART-controlled subjects and may represent a step forward in the comprehension of the physiopathology and the treatment of the HIV infection [8, 21]. In contrast to the anticipated outcome, no significant difference was observed in 16S rDNA rates according to HCV clinical stage or immunosuppression level [2]. On the other hand, univariate analysis showed that LBP was significantly higher in patients with higher HIV viral load (p = 0.03) and sCD14 was significantly higher in patients with a CDC clinical stage C (p<0.001) (Fig 3). Surprisingly, I-FABP was significantly higher in patients with an undetectable HIV viral load (p = 0.002) (Fig 3). None of the three markers were associated with CD4+ cells count, HCV genotype or the METAVIR score. The multivariate linear regression analyses confirmed the findings of the univariate comparisons. Plasma sCD14 was significantly higher in patients with an advanced CDC stage (Stage B vs A: RGM = 1.15, CI95% = [1.01:1.31], p = 0.04 and stage C vs A: RGM = 1.21, CI95% = [1.04:1.41], p = 0.02). Plasma I-FABP was significantly higher in patients with undetectable HIV viral load (p<0.001). Plasma I-FABP was significantly higher in patients with CD4+ cells count below 350/μL as compared to patients with CD4+ cells count higher than 350/μL (RGM = 1.56, CI95% = [1.04:2.36], p = 0.04). The phenomenon of a higher I-FABP level in patients with undetectable HIV viral load and also in immunosuppressed patients has not previously been described. Mechanisms explaining this high rate of I-FABP remain to be established.

## Conclusions

In conclusion, indirect markers of MT (LBP, sCD14S) were significantly higher in co-infected patients than in healthy controls, reflecting a persistence of immune activation correlated with the HIV evolution (clinical stage, HIV viral load) in these patients. The levels of these markers were not correlated to HCV evolution (persistence or chronicity of HCV, HCV viral load and METAVIR score,). However, our findings show that in these patients, the level of a direct marker of MT (plasma 16S rDNA) seems to be linked to the duration of HIV infection, demonstrating that HIV alone is responsible for MT. In contrast, a high level of I-FABP has been shown in patients with undetectable viral load, possibly linked to the intestinal viral reservoir. Further work is needed to demonstrate the link between undetectable viral load and intestinal permeability.

## Supporting information

S1 Table Full characteristics of study population. ^a^Centers for Disease Control and Prevention.
^b^Outcome after anti-HCV therapy 1: Sustained viral response (VR); 2: Discontinued treatment; 3: No response; 4: Partial VR; 5: Relapse; 6: In treatment.
^c^Lipopolysaccharide (LPS)-binding protein.
^d^soluble CD14.
^e^intestinal fatty acid binding protein.
^f^ No data.
^g^ Not applicable.
(XLS)
